# Peptipedia v2.0: a peptide sequence database and user-friendly web platform. A major update

**DOI:** 10.1093/database/baae113

**Published:** 2024-11-08

**Authors:** Gabriel Cabas-Mora, Anamaría Daza, Nicole Soto-García, Valentina Garrido, Diego Alvarez, Marcelo Navarrete, Lindybeth Sarmiento-Varón, Julieta H Sepúlveda Yañez, Mehdi D Davari, Frederic Cadet, Álvaro Olivera-Nappa, Roberto Uribe-Paredes, David Medina-Ortiz

**Affiliations:** Departamento de Ingeniería en Computación, Universidad de Magallanes, Av. Pdte. Manuel Bulnes 01855, Punta Arenas 6210427, Chile; Centre for Biotechnology and Bioengineering, CeBiB, Universidad de Chile, Avenida Beauchef 851, Santiago 8320000, Chile; Departamento de Ingeniería en Computación, Universidad de Magallanes, Av. Pdte. Manuel Bulnes 01855, Punta Arenas 6210427, Chile; Departamento de Ingeniería en Computación, Universidad de Magallanes, Av. Pdte. Manuel Bulnes 01855, Punta Arenas 6210427, Chile; Centro Asistencial de Docencia e Investigación, CADI, Universidad de Magallanes, Av. Los Flamencos 01364, Punta Arenas 6210005,Chile; Centro Asistencial de Docencia e Investigación, CADI, Universidad de Magallanes, Av. Los Flamencos 01364, Punta Arenas 6210005,Chile; Escuela de Medicina, Universidad de Magallanes, Av. Pdte. Manuel Bulnes 01855, Punta Arenas 6210427, Chile; Centro Asistencial de Docencia e Investigación, CADI, Universidad de Magallanes, Av. Los Flamencos 01364, Punta Arenas 6210005,Chile; Centro Asistencial de Docencia e Investigación, CADI, Universidad de Magallanes, Av. Los Flamencos 01364, Punta Arenas 6210005,Chile; Facultad de Ciencias de la Salud, Universidad de Magallanes, Av. Pdte. Manuel Bulnes 01855, Punta Arenas 6210427, Chile; Department of Bioorganic Chemistry, Leibniz Institute of Plant Biochemistry, Weinberg 3, Halle 06120, Germany; PEACCEL, Artificial Intelligence Department, AI for Biologics, Paris 75013, France; Centre for Biotechnology and Bioengineering, CeBiB, Universidad de Chile, Avenida Beauchef 851, Santiago 8320000, Chile; Departamento de Ingeniería en Computación, Universidad de Magallanes, Av. Pdte. Manuel Bulnes 01855, Punta Arenas 6210427, Chile; Centre for Biotechnology and Bioengineering, CeBiB, Universidad de Chile, Avenida Beauchef 851, Santiago 8320000, Chile; Departamento de Ingeniería en Computación, Universidad de Magallanes, Av. Pdte. Manuel Bulnes 01855, Punta Arenas 6210427, Chile; Centre for Biotechnology and Bioengineering, CeBiB, Universidad de Chile, Avenida Beauchef 851, Santiago 8320000, Chile

## Abstract

In recent years, peptides have gained significant relevance due to their therapeutic properties. The surge in peptide production and synthesis has generated vast amounts of data, enabling the creation of comprehensive databases and information repositories. Advances in sequencing techniques and artificial intelligence have further accelerated the design of tailor-made peptides. However, leveraging these techniques requires versatile and continuously updated storage systems, along with tools that facilitate peptide research and the implementation of machine learning for predictive systems. This work introduces Peptipedia v2.0, one of the most comprehensive public repositories of peptides, supporting biotechnological research by simplifying peptide study and annotation. Peptipedia v2.0 has expanded its collection by over 45% with peptide sequences that have reported biological activities. The functional biological activity tree has been revised and enhanced, incorporating new categories such as cosmetic and dermatological activities, molecular binding, and antiageing properties. Utilizing protein language models and machine learning, more than 90 binary classification models have been trained, validated, and incorporated into Peptipedia v2.0. These models exhibit average sensitivities and specificities of 0.877±0.0530 and 0.873±0.054, respectively, facilitating the annotation of more than 3.6 million peptide sequences with unknown biological activities, also registered in Peptipedia v2.0. Additionally, Peptipedia v2.0 introduces description tools based on structural and ontological properties and user-friendly machine learning tools to facilitate the application of machine learning strategies to study peptide sequences.

**Database URL**: https://peptipedia.cl/

## Introduction

Peptides are versatile biomolecules that can be synthetic or found in natural sources and are attractive candidates for therapeutic applications [1–3]. Peptides play crucial roles in numerous biological processes. Their functions are diverse, serving as structural components, enzymatic inhibitors, cell-penetrating agents, hormones, host defence molecules, and neurotransmitters [3]. Additionally, peptides act as cell surface receptors [4] and are integral to drug delivery applications [5].

Peptide drugs present several potential advantages over traditional small-molecule drugs, such as increased selectivity, affinity, efficacy, and safety, along with reduced toxicity and immunogenicity [6]. However, their widespread clinical application is hindered by challenges, including a short half-life, limited oral bioavailability, and susceptibility to plasma degradation [7].

The therapeutic market started 100 years ago using insulin to treat type 1 diabetes [8, 9]. To date, over 100 peptide drugs are currently available in the global market for various diseases, including human immunodeficiency virus (HIV) infection, chronic pain, metabolic disorders, and infectious diseases [3, 10, 11]. The global therapeutic peptide market reached a value of US$42.05 billion in 2022 [12]. Due to research efforts, developed technology, and the investment of pharmaceutical companies, it is expected that peptide drug discovery and development will continue to expand in the upcoming years, foreseen to reach US$68.6 billion by 2030 (with a compound annual growth rate 6.3% from 2023 to 2030) [1, 12–14].

The lack of structured and specialized peptide repositories drove the development of Peptipedia, an integrated peptide database, and its user-friendly web platform [15]. Peptipedia v1.0 aggregated over 92 000 peptide sequences and more than 75 000 peptides with documented biological activities from 30 diverse data sources. This centralized repository not only offered a comprehensive collection of peptide data but also included valuable tools such as a physicochemical properties estimator, statistical characterization of amino acid sequences, and bioinformatics resources like sequence alignment to enhance peptide research. Moreover, Peptipedia v1.0 incorporated machine learning-based binary classification models to predict potential biological activities, including antimicrobial, antiviral, and antibacterial properties. Integrating these features in a single platform significantly advanced the accessibility and analysis of peptide-related information, underscoring the importance of an integrated peptide database for the scientific community.

Before Peptipedia v1.0, peptide information was widely spread in diverse databases such as Database of Antimicrobial Activity and Structure of Peptides, Erop-Moscow, Linking Antimicrobial Peptides database (LAMP), Data Repository of Antimicrobial Peptides (DRAMP), and Structurally annotated therapeutic peptides database (SATPdb) [16–20]. Peptide-related databases comprehend from a very specialized topic, i.e. blood-brain barrier peptides [21], quorum sensing peptides [22], anti-angiogenic peptides predictor [23], and bacteriocin peptides [24], to general databases, i.e. the Universal Protein Resource (UniProt) [25]; LAMP2, a generic antimicrobial peptide database [18]; DRAMP, a data repository of antimicrobial peptides [19]; and SATPdb, a database of structurally annotated therapeutic peptides [20].

Despite the availability of numerous databases, integrating information between them remains challenging. Peptides are usually classified by just one functional biological activity. However, moonlight peptides have two or more known activities within the same domain [26], and identifying the potential multiple activities of a peptide is relevant for biotechnological and pharmaceutical industries [27, 28].

Peptipedia is a pivotal tool for peptide research, designed to extract, consolidate, organize, and curate information from multiple databases, significantly enhancing knowledge in the field. While Peptipedia v1.0 served as a powerful resource, its development revealed several areas for improvement. Firstly, the database could benefit from a substantial increase in the number of records and a broader scope of information related to each peptide. Secondly, the classification of biological activities in v1.0 was limited, often relegating many peptide activities to a generic ‘other’ category, reducing the data’s specificity and utility. Lastly, the lack of an automated system for updating newly generated data limited the platform’s ability to stay current and fully realize its potential.

The experience gained from Peptipedia v1.0 was instrumental in shaping the second version of the database. By addressing these identified challenges, Peptipedia v2.0 offers a more comprehensive and dynamic knowledge platform. It provides enhanced data integration, expanded records, a more refined classification system for biological activities, and an automatic updating mechanism. These advancements ensure that Peptipedia continues to serve as a cutting-edge resource for researchers, facilitating deeper insights and fostering innovation in peptide research.

This work presents a significant update to the Peptipedia database, incorporating over 3.8 million peptide sequences from more than 70 data sources. Over 100 000 peptides are documented as having functional biological activity based on the literature. The categories and subcategories of the biological activity tree were meticulously examined and reorganized, with new activities such as anti-ageing, cytokine, and molecular binding included. An automatic update system was also implemented, and the advanced search and downloading functionalities were enhanced.

In Peptipedia v2.0, various types of information have been linked to each peptide sequence, including functional domains [29], gene ontology annotations [30, 31], secondary structure information [32], and 3D structures available through Protein Data Bank (PDB) [33] and AlphaFold [21, 34] databases.

Moreover, Peptipedia v2.0 incorporates data on physicochemical property descriptors, patents, and previous publications. Bioinformatic enrichment analysis and statistical evaluation tools have also been integrated. Furthermore, the functional classification models have been updated, enhancing the precision of individual models and improving their generalization. These trained classification models evaluated over 3.7 million peptide sequences, predicting their functional biological activities.

Finally, Peptipedia v2.0 includes machine learning tools for users to analyse their datasets and build their models. These tools encompass protein language models and classical amino acid coding methods for numerical representation strategies, predictive model training through traditional supervised learning algorithms, pattern recognition using unsupervised learning approaches, and sequence similarity networks combined with community detection techniques for pattern recognition. With these enhancements, users can gain deeper insights and uncover novel characteristics from their datasets. Consequently, all these new tools and updates establish Peptipedia v2.0 as one of the most comprehensive, thoroughly processed, and tractable repositories of peptides currently available.

## Methodologies and implementation strategies

### Collecting and processing peptide sequences

This work searched databases, datasets, and public repositories related to the study of peptides to update and increase the number of records in the Peptipedia v2.0 database. Different keywords were used to collect the data sources in Google Scholar, like ‘peptides’, ‘AMPs’, ‘neuropeptides’, ‘anticancer peptides’, ‘nutraceutical peptides’, and ‘signal peptides’. Besides, generic protein databases such as UniProtKB [25] and RCSB PDB [35] were incorporated (see section S1 of Supplementary data for more details).

Information related to the characteristics of the reported peptide sequences, including their biological activities, descriptions, experimental information, and related publications or patents, was downloaded from all data sources.

Then, Python scripts were implemented to process the raw data downloaded from the data sources and transform the information for loading into the Peptipedia v2.0.

A length filter was made, containing only peptides with a length equal to or less than 150 residues and higher than three residues. Also, the collected peptides were classified as canonical (with only the 20 natural amino acids) or non-canonical peptides. Then, a semantic analysis was generated to recognize the biological activity of the peptide sequence through the available description in the data sources.

Finally, a loader Python script was implemented to load the register in the Peptipedia v2.0, developing a scalable Extract, Transformation, and Load (ETL) strategy for each utilized data source.

### Peptide descriptions and enrichment analysis implementation

Each peptide incorporated in the Peptipedia v2.0 was characterized using the peptide descriptor service and enrichment analysis system.

First, the modlAMP tool [36] was used to calculate the physicochemical properties of canonical peptide sequences. Then, the standalone version of RaptorX-Property tool [32] was employed to predict the secondary structure of all records available in Peptipedia v2.0.

Through homology mechanisms, enrichment analysis was performed for all canonical peptides using the MetaStudent tool [37]. MetaStudent allows the assignment of gene ontology terms from different sources, such as molecular function, cellular localization, and biological process.

Once the gene ontology terms prediction was completed, the results were filtered using the data returned probability score, selecting only results with a probability higher than .5 as elements to incorporate as a descriptor of the analysed peptide sequence. Finally, PfamScan tool [38] was used to estimate protein domain families for all peptides registered in the Peptipedia v2.0.

### Training predictive models

This work implements binary classification models to identify peptide sequences’ functional biological activities by combining embedding representation through pretrained models with classic supervised learning algorithms [39, 40].

A binary dataset was constructed for each biological activity identified in the Peptipedia v2.0. The following steps were used to build the datasets: (i) peptides exhibiting the target activity were collected to generate positive examples, (ii) peptides without the target activity were collected to create the negative examples, including, where possible and if the information was available, experimentally validated negative examples. This was especially developed for peptides with antimicrobial, antiviral, or antibacterial activity. In addition, the same peptides used as negative examples in previously reported works have been used as negative examples for different activities [41–44],; (iii) the CD-Hit tool [45] was applied to remove redundancy in each category using a homology percentage of 90% and the rest of the configuration parameters by default [40, 46], and the representative sequences were employed to rebuild the binary classification dataset, (iv) an undersampling strategy was applied to balance the dataset by randomly removing negative examples, and (v) different pretrained models were then used to generate embeddings, representing peptide sequences numerically for training the classification models [47] (see more details in Section S2 of Supplementary data).

Activities with fewer than 50 peptides were excluded because they are classified as Low-*N* datasets, and more specialized strategies, such as transfer learning, semi-supervised approaches, and contrastive learning methods, are required to train predictive models using these types of datasets [48].

Each dataset built is divided into training and validation, using an 80:20 proportion. Nine supervised learning algorithms, including Decision Tree, Random Forest, and XGBoost, were used to train classification models using the training dataset. A *K*-fold cross-validation (*k* = 10) was performed to prevent overfitting. The validation dataset was then used to assess model performance using classical metrics such as precision, recall, accuracy, and *F*-score [49]. The training process, including the division between the training and validation datasets, training under *k*-fold cross-validation, and evaluating the performances using the metrics summarized in section S6 of Supplementary Materials were repeated *n* = 30 times to demonstrate the generalization capacity of the trained strategies, as proposed in our previous work [40].

The combination of supervised learning algorithm and embedding representation is selected using the performances obtained during the training and validation process, applying the following criteria: (i) highest performance during the training stage, (ii) highest performance during the validation stage, and (iii) lowest differences between training and validation to reduce the overfitting. Four classic metrics were employed to evaluate the trained models during the training and validation process, including accuracy, precision, recall, and *F*-score (see more details in Section S6 of Supplementary data)

Finally, the models were exported in joblib format for integration into the Peptipedia v2.0.

### Implementation strategies and availability system

The database available in Peptipedia v2.0 was designed based on a relational schema. PostgreSQL manages all operations over the database. All queries performed to the database are managed through an application programming interface implemented using the Flask framework version 2.3 and SQLAlchemy version 2.

The ETL system, description process, and enrichment analysis strategies were implemented using Python version 3.9. Moreover, each tool and service available in Peptipedia v2.0 were implemented using Python programming version 3.9.

All binary classification models were implemented using Python v3.9.17 and the supervised learning module available on the DMAKit Python library [50]. Moreover, the embedding representation for the peptide sequences was performed using the bioembedding library [47].

Finally, the user-friendly web platform in Peptipedia v2.0 was implemented using the React framework as the front end. The system was deployed using Podman into a public server with AlmaLinux 9 as the operative system. Its hardware characteristics are eight vCPU Cores, 64 GB RAM, 500 GB NVMe, and 32 TB Traffic.

## Results and discussion

Peptipedia v2 is a user-friendly web platform designed to facilitate the study of peptide sequences using advanced bioinformatics tools and machine learning strategies. This updated version boasts a database of over 100 000 peptides sourced from > 70 databases. Various physicochemical and thermodynamic properties characterize each peptide.

New features in Peptipedia v2 include enrichment analysis through Gene Ontology terms, secondary structure predictions, and functional domain evaluations, providing more comprehensive information on each peptide sequence. The functional biological activity tree has been enhanced to include more specific cosmetics, dermatology, taste, and molecular binding activities.

Additionally, the platform now implements over 90 binary classification models for biological activity classification, utilizing embedding representations from pretrained models and traditional supervised learning algorithms. Numerous tools, such as advanced search capabilities, peptide sequence downloading, physicochemical characterization, and functional biological activity classification, have been updated or newly incorporated.

**Figure 1.**
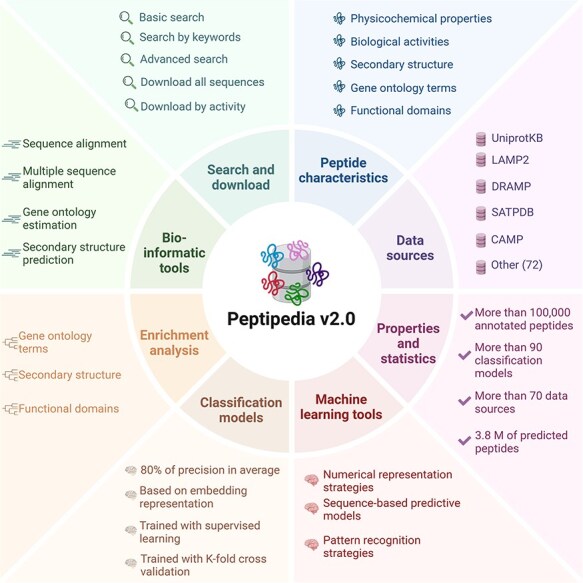
Overview of Peptipedia v2.0. This new version of Peptipedia includes > 100 000 peptides registered with functional biological activities, extracted from > 70 data sources. Different physicochemical properties and enrichment analyses were addressed to characterize the peptides collected. The services and functionalities were updated, incorporating gene ontology and functional domain predictions, secondary structure evaluation, and physicochemical properties estimation. Besides, > 90 functional biological activity classification models were implemented, combining embedding representation through pretrained models and supervised learning algorithms. Finally, customizable machine learning pipelines could be implemented by employing the machine learning tools in Peptipedia v2.0 to facilitate the application of machine learning techniques to study peptide sequences.

Moreover, Peptipedia v2.0 includes an integrative machine learning pipeline to streamline the development of sequence-based predictive models and pattern recognition. An overview of Peptipedia v2.0 is presented in Figure 1, highlighting the most relevant data sources, peptide sequence information, bioinformatics tools, and machine learning applications.

### New data sources, biological activity tree, and peptide sequences

**Figure 2.**
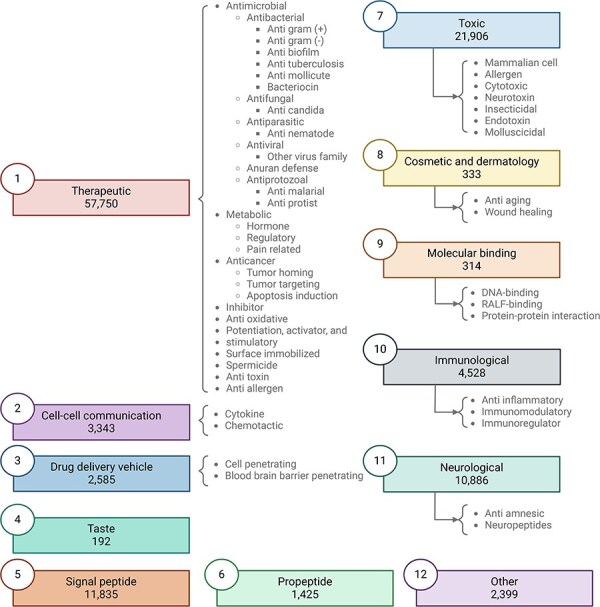
Updated functional biological activity tree implemented in Peptipedia v2.0. In this work, we have updated the functional biological activity tree to include new activities related to taste, molecular binding, and cosmetics and dermatology. Additionally, various antiviral activities have been added, specifically targeting virus families and different mechanisms, with a particular focus on HIV. We have also incorporated peptides associated with therapeutic functions, such as inhibitors, antitoxins, antiallergens, and spermicides. Peptides with toxic effects have also been included, constituting a significant part of the newly reported peptides in Peptipedia v2.0. Lastly, the category of peptides classified as ‘other’ has been updated to encompass diverse biological activities unrelated to the main proposed activities, such as participation in photosynthesis and antibarnacle properties.

Peptipedia v2.0 includes 76 data sources, comprising databases, repositories, and datasets (see Section S1 of Supplementary data for more details). These data sources are dedicated to collecting and retrieving peptide sequences alongside their corresponding biological activities. The data sources used in this Peptipedia v2.0 database increased by over 100% in the number of data sources compared to the initial version of Peptipedia [15]. A comprehensive update to the biological activity tree has been executed alongside expanding data sources. Figure 2 summarizes the updated functional biological activity tree in Peptipedia v2.0.

In this new version of the biological activity tree, the activities are organized into 11 primary biological activities at the first level of the tree. Besides, the updated tree has incorporated an additional activity called ‘other’. While maintaining activities associated with therapeutic and immunological effects, this version introduces additional biological activities such as peptides inducing toxic effects, taste peptides, and those with cosmetic and dermatological activities (see Table 1 and Section S3 of Supplementary data for more details).

**Table 1.** Summary of activities classified in the first level of the updated functional biological tree

Biological activity	Definition	Records	Data sources
Therapeutic	Therapeutic peptides are molecules, whether designed or natural, with medical applications that aim to treat or alleviate diseases by exerting various bodily functions.	57 750	51
Cell–cell communication	Peptides involved in cell-to-cell communication, which can influence cell signaling and cell-mediated immune responses.	3343	6
Drug delivery vehicle	These peptides function as carriers that transport and deliver drugs in a specific manner to target cells or tissues. They can target specific receptors on cells to release their therapeutic cargo.	2858	11
Taste	A short chain of amino acids that elicits a specific taste sensation when it interacts with taste receptors on the tongue. Taste peptides can evoke a range of tastes, including sweet, bitter, salty, sour, and umami.	192	2
Signal peptide	Peptides that direct proteins to specific cellular locations used in protein secretion and intracellular transport processes, which may affect cellular communication and protein-mediated immune responses	11 835	9
Propeptide	Inactive peptides that are converted to active peptides by processing, act as a precursor to a protein, and are removed during processing, which can influence biological activity and protein regulation.	1424	2
Toxic	Peptides that possess harmful properties and can induce toxic effects in biological systems. These peptides may interfere with normal cellular functions, alter metabolic processes, or cause damage to cells and tissues.	21 906	20
Cosmetic and dermatology	Small-chain amino acid peptides are used in skin care and cosmetic products. These peptides are designed to address various skin-related concerns such as wrinkles, fine lines, loss of elasticity, and other signs of skin ageing.	333	5
Molecular binding	Peptides that bind specifically to molecules, such as proteins, RNA, or DNA, which may influence the regulation of biological processes and immune responses.	314	3
Immunological	Peptides have properties that influence the body#x2019;s immune response and interact with components of the immune system. They can modulate the function of immune cells and play a role in regulating and modulation of the immune response.	4258	11
Neurological	Essential peptides in nervous system signalling, influencing cellular communication and functions such as memory and neuronal regeneration.	10 886	5
Other	Peptides with other activities non-generalizable with activities in the first level of the tree, including activities like reductant, pore-forming, or ribonucleoprotein	2399	10

In Peptipedia v2.0, only 2399 peptides were categorized as ‘other’. These peptides exhibit biological activity but cannot be classified within the biological activities described in the implemented tree. Additionally, only 1034 peptides—approximately 1% of those registered in this updated version—lack reported activities. This highlights substantial improvements in peptide description and functional understanding compared to the first version reported in Peptipedia [15].

The updated biological activity tree aims to simplify peptide classification by minimizing ambiguity and facilitating more explicit sequence reporting. Integrating more data sources has resulted in a significant increase in sequences with reported biological activities. Peptipedia v2.0 now incorporates over 100 000 sequences with reported biological activities, representing a surge of over 40% from its initial release [15]. This positions Peptipedia v2.0 not only as the largest repository of peptide sequences but also as the most extensive collection of peptides with therapeutic and antimicrobial activities, exceeding traditional databases such as Erop Moskow [51] and SATPDb [52]. Peptipedia v2.0 significantly surpasses traditional peptide databases in both the quantity and comprehensiveness of its records. For instance, databases such as LAMP2 [53], DBAMP [54], and SATPDb [52] collectively document over 20 000 antimicrobial peptides. In contrast, Peptipedia v2.0 alone registers > 40 000 antimicrobial peptides, demonstrating its superior coverage. Similarly, when considering specialized repositories for antiviral peptides, databases like AVPiden [55], Antiviral Peptides Database [56], and Dravp [57], each catalogs fewer than 4000 antiviral peptides, whereas Peptipedia v2.0 boasts a record of over 5500 such peptides. The same trend is observed with antifungal peptides: databases such as Ampfun [58] and LAMP2 [53] report fewer than 7000 antifungal peptides, covering only about 65% of the peptides collected in Peptipedia v2.0. This comparison highlights Peptipedia v2.0 as a more extensive and valuable resource for researchers in the peptide field.

### Peptipedia incorporates an autonomous updating pipeline

The significant increase in records poses a challenge in maintaining updates, integrating new sequences, and ensuring data integrity. Extract, Transform, and Load systems (ETL) have been designed and implemented to address these database maintenance challenges.

**Figure 3.**
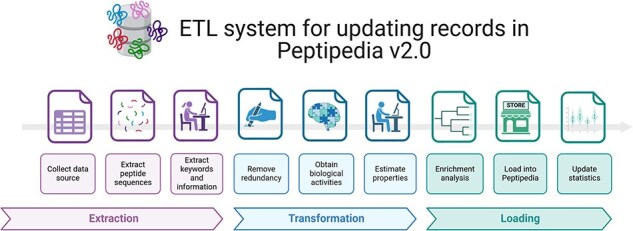
ETL system implemented for processing a data source and loading the collected information into Peptipedia DB. The ETL system implemented in Peptipedia facilitates the process of a new data source to extract and load the information into Peptipedia DB. First, the different information is extracted from the data source, including the peptide sequence, references, keywords, and relevant properties described in the data source. Then, two transformation processes are applied to collect raw data to facilitate the characterization of the peptide sequences and the identification of the functional biological activity. Also, an enrichment analysis is run to obtain gene ontology terms and functional domains. Finally, the load process implies inserting or updating the records in the Peptipedia DB, a statistical analysis, and a summary process of the ETL execution.

Figure 3 provides an overview of the implemented ETL process for processing a data source. The data source is initially used to extract pertinent information, including peptide sequences, function-associated keywords, literature references, patents, and pharmacological and physicochemical properties. Additionally, metadata is collected and saved for upload into Peptipedia v2.0.

After data collection and extraction of relevant information, a redundancy evaluation is conducted to remove duplicate elements. The data are then processed to determine biological activity using the functional biological activity tree implemented in Peptipedia v2.0. Peptides are classified as either canonical or noncanonical based on their sequences. For peptides categorized as canonical, physicochemical and thermodynamical properties are estimated using the ModLAMP library [36].

Next, an enrichment analysis, including Gene Ontology prediction and functional domain analysis through the Pfam tool, is applied to all canonical peptides.

Once the peptide sequences are processed, the loading process begins and the peptide sequences are inserted or updated in Peptipedia v2.0. Subsequently, the metadata, statistics, and summary records in Peptipedia v2.0 are updated, completing the process (see Section S7 of Supplementary data for a database description and a visualization of implemented materialized views in Peptipedia v2.0).

An extraction process was developed for each data source to update Peptipedia v2.0. This implementation is critical due to the unique rules and strategies governing the storage and deployment of information within each data source.

Working with each data source individually simplifies the update process, facilitating individualized execution of record updates within Peptipedia v2.0. This flexibility enables tailored configurations for updates based on the update periods stipulated by the data sources. For instance, databases such as UniProt or PDB undergo monthly updates, whereas databases like SATPdb receive annual updates. Conversely, data sources requiring regular updates are included in the Peptipedia v2.0 update process. Finally, the ETL system can be executed automatically for each employed data source or manually in the case of new databases incorporated into Peptipedia v2.0.

### Improving functionalities and implementing new bioinformatics tools

Peptipedia v2.0 introduces different tools to facilitate the study of peptide sequences. Firstly, enrichment analysis methods have been integrated, leveraging the predictive capabilities of Gene Ontology terms [31] and protein functional domains through the Pfam tool [38]. Secondly, the prediction of secondary structures and essential structural properties, such as solvent accessibility, is simplified by integrating the RaptorX-Property tool [32].

Furthermore, new tools and methods have been implemented to facilitate the application of machine learning algorithms to study peptide sequences. Different numerical representation strategies have been incorporated to simplify the preprocessing of peptide sequences. These strategies encompass coding amino acids based on their physicochemical properties and utilizing learning representations derived from pretrained models [47, 59].

The updated machine learning tool now effectively trains predictive models using diverse supervised learning algorithms, including the Gaussian Process, ensemble methods, support vector machines, and nearest neighbour algorithms. Besides, the metrics used to evaluate the training process’s performance have been refined, encompassing performance metrics like Precision, recall, and F1-score for classification tasks and root mean squared error for regression tasks. Both models trained, dataset processed, and summary training process could be downloaded to facilitate using the generated model in a local environment.

Moreover, Peptipedia v2.0 offers pattern recognition capabilities through clustering strategies based on unsupervised learning algorithms, sequence similarity networks, and community detection methods. Peptipedia v2.0 facilitates evaluating the generated groups by applying performance metrics like the Calinski–Harabasz index and the Silhouette coefficients. These algorithms generate cohesive groups characterized using statistical analyses and enrichment tools.

Finally, Peptipedia v2.0 has updated bioinformatic tools for sequence characterization. These include physicochemical property analyses, sequence alignments against the Peptipedia database, and the integration of multiple sequence alignments and statistical description methodologies for groups of protein sequences, which have been implemented to support the study of peptide sequences.

### New classification models and predicting nonannotated peptide sequences

The enhancement of the biological activity tree and the expansion of peptide sequences necessitated updating the binary classification models initially developed for Peptipedia. Following the sequence-based approach detailed in the Methods section, this work used 98 biological activities to train binary classification models.

We evaluated 90 combinations of numerical representation strategies and supervised learning algorithms for each biological activity, resulting in over 8 800 trained models. On average, the specificity is $0.807 \pm 0.084$ and the sensitivity is $0.813 \pm 0.082$ for the evaluated models (see Section S4 of Supplementary data for more details). Algorithms like ExtraTrees and Random Forest demonstrated the highest sensitivity and specificity, with values exceeding $0.83 \pm 0.06$. In contrast, Decision Tree-based models showed the lowest performance, with sensitivity and specificity values below $0.74 \pm 0.08$. Regarding numerical representation strategies, models trained using the pretrained models ProTrans t5 xlu50 and ProTrans t5 Uniref exhibited the highest performance, with sensitivity and specificity values above $0.82 \pm 0.08$. Conversely, the ProTrans t5 XLNET model showed the lowest performance, with sensitivity and specificity values below $0.78 \pm 0.08$ (see Section S4 of Supplementary data for more details).

The most effective combinations of numerical representation strategies and supervised learning algorithms involved ProTrans t5 Uniref or ProTrans t5 xlu50 with ExtraTrees, achieving average sensitivity and specificity values exceeding $0.85 \pm 0.06$. ProTrans t5 XLNET with Decision Tree was the least effective combination, with average sensitivity and specificity values below $0.72 \pm 0.09$.

Based on the sensitivity and specificity performance analysis and the criteria described in the Methodology section, 98 binary classification models were selected from the evaluated combinations. The chosen models achieved average sensitivities and specificities of $0.877 \pm 0.053$ and $0.873 \pm 0.054$, respectively. Combinations such as ProTrans t5 Uniref with ExtraTrees, ProTrans t5 Uniref with Gaussian Process, and ProTrans t5 xlu50 with ExtraTrees were the most frequently selected, representing 25% of all evaluated activities. In contrast, combinations like Esm1B with Adaboost, Esm1B with Random Forest, and ProTrans t5 XLNET with XGBoost were the least frequently selected, representing only 0.9% of the total explored activities (see Section 4 of Supplementary data for more details).

A ranking of the best models (see Table 2) and the models with the lowest performance (see Table 3) based on the Matthews correlation coefficient (MCC) metric was generated. The top models were associated with various target virus activities, including anti-human parainfluenza virus and anti-Andes virus, different virus families like *Bunyaviridae* and *Hantaviridae*, and activities such as DNA-binding, neurotoxin, and transit. In contrast, the models with the lowest MCC values were related to activities such as Cosmetic and Dermatology, Blood-Brain Barrier Penetration, and Anticancer. However, despite being classified as the lowest-performing models, their MCC values were higher than 0.5, and they achieved precision averages over 75%, demonstrating high generalization and robustness of the implemented strategies.

**Table 2.** Best models based on MCC performances for binary classification of functional biological activities.

Activity	Algorithm	Encoder	MCC	Accuracy	F1-score	Precision	Recall
Anti-human parainfluenza virus	Random orest	Prottrans t5bdf	0.999	0.999	0.999	0.999	0.999
*Anti-Bunyaviridae*	XGB	Prottrans t5bdf	0.974	0.986	0.986	0.987	0.986
Anti-sin Nombre virus	Random Forest	Prottrans t5bdf	0.974	0.986	0.986	0.987	0.986
DNA-binding	Bagging	Prottrans t5 uniref	0.972	0.986	0.986	0.986	0.986
Gluten immunogenic and celiac toxic	XGB	Prottrans xlnet	0.970	0.985	0.985	0.985	0.985
*Anti-Hantaviridae*	AdaBoost	Prottrans xlnet	0.962	0.980	0.980	0.981	0.980
Anti-Andes virus	Random Forest	Prottrans t5bdf	0.961	0.980	0.980	0.981	0.980
Neurotoxin	Gradient Boosting	Esm1b	0.948	0.974	0.974	0.974	0.974
Transit	K Neighbors	Prottrans t5 xlu50	0.943	0.971	0.971	0.972	0.971
Cytokine	Gaussian Process	Prottrans t5 uniref	0.910	0.955	0.955	0.955	0.955

**Table 3.** Models with the lowest MCC performances for binary classification of functional biological activities.

Activity	Algorithm	Encoder	MCC	Accuracy	F1-score	Precision	Recall
Anticancer	K Neighbors	Prottrans t5 uniref	0.607	0.800	0.799	0.806	0.800
*Anti-Retroviridae*	Gaussian Process	Prottrans t5 xlu50	0.604	0.802	0.802	0.802	0.802
*Anti-methicillin-resistant S. aureus*	Bagging	Esm1b	0.602	0.806	0.806	0.806	0.806
Cytotoxic	Gaussian Process	Prottrans t5 uniref	0.599	0.798	0.797	0.802	0.798
Blood brain barrier penetrating	Gradient Boosting	Prottrans t5 uniref	0.591	0.796	0.796	0.796	0.796
Anti-angiogenic	Extra Trees	Prottrans t5 uniref	0.579	0.789	0.788	0.790	0.789
Anti-biofilm	Extra Trees	Esm1b	0.578	0.788	0.788	0.789	0.788
Anti-influenza virus	Extra Trees	Prottrans xlnet	0.576	0.790	0.789	0.790	0.790
Cosmetic and dermatology	Random Forest	Prottrans t5bdf	0.576	0.785	0.784	0.790	0.785
Anti-Candida	Extra Trees	Prottrans t5 xlu50	0.505	0.753	0.753	0.754	0.753

Finally, the trained classification models evaluated 3.8 million sequences without reported biological activity incorporated into Peptipedia v2.0. Table 4 summarizes the classification results for the biological activities at the first level of the updated biological activity tree. Except for peptides with Molecular Binding activity, all biological activities saw an increase in records by more than 100%. Notably, categories such as therapeutics, signal peptides, propeptides, immunological, and neurological activities increased by more than 600 000 peptides. This suggests a tendency for immunological activity and propeptides among previously uncharacterized peptides. Additionally, more than 800 000 peptides showed potential therapeutic activity, indicating that they could serve as alternatives to traditional drug-based compounds. However, further evaluation of these peptides is necessary, including toxicity and immunogenicity assessments and structural analyses to understand their properties compared to previously validated therapeutic peptides [7].

**Table 4.** Summary classified unknown peptides used the trained classification models

#	Activity (first level in tree)	Labelled peptides	Predicted peptides
1	Therapeutic	57 750	837 036
2	Cell–cell communication	3343	6126
3	Drug delivery vehicle	2585	17 984
4	Taste	192	97 405
5	Signal peptide	11 835	851 175
6	Propeptide	1425	1 215 114
7	Toxic	21 906	112 584
8	Cosmetic and dermatology	333	74 956
9	Molecular binding	314	196
10	Immunological	4528	1 001 216
11	Neurological	10 886	715 027

### A new and modern face for the Peptipedia web platform

**Figure 4.**
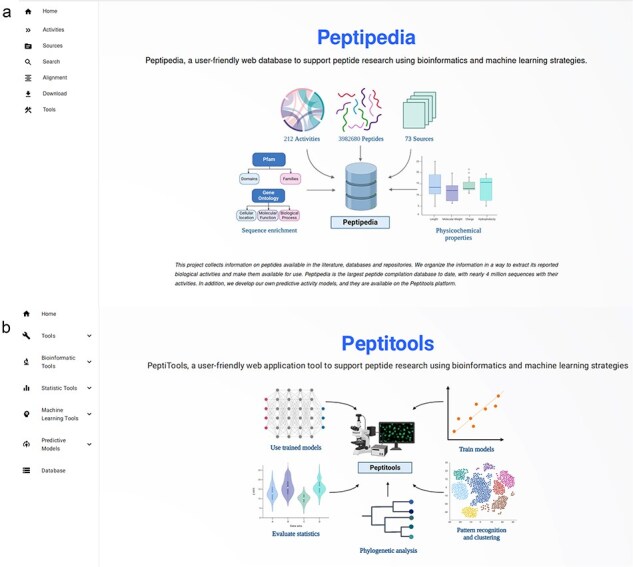
Homepage for Peptipedia DB and Peptitools, the two main services implemented into Peptipedia. (a) Homepage generated to access on Peptipedia DB. This homepage has (i) the full menu with all functionalities available on the system, (ii) a schematic representation of the implemented workflow to process collected data from data sources, (iii) a statistical description of register information, and (iv) relevant information on the project. (b) Homepage generated to access on PeptiTools. This homepage includes a schematic representation of implemented tools and a full menu to access a desirable tool.

In this new version of Peptipedia, a visual restructuring of the web platform was designed and implemented. Using web development technologies based on React, a new front-end was developed. The new Peptipedia platform separates the tools for visualizing the database and their respective data searching and downloading options, including Fasta and Comma separated value format files. The separation of tools and databases allows for optimizing response times for each user-generated action (See Figure 4 for a schematic representation and Section S5 of Supplementary data for more details).

Upon accessing the Peptipedia web platform via its access link, the user will first see the Peptipedia homepage (See Figure 4a). This homepage features information about the database, a statistical summary of the information recorded in Peptipedia, a diagram illustrating how data are collected and processed, and information describing the workgroup and the project (see more details in Section S5 of Supplementary data for more information).

The Peptipedia menu includes the main accesses to the platform, including activity analysis, data sources, direct download systems, and the search engine. The latest version of the sequence search engine in Peptipedia facilitates the application of different filters to customize the type of information identification. Query results have also been optimized through the generation of materialized views. The visualization of the results has been updated to present the characteristics of the identified sequences in a more user-friendly manner, incorporating all existing information in the platform (see Section S5 of Supplementary data for more details).

From the perspective of the computational tools implemented in Peptipedia, a new platform called PeptiTools has been developed (see Figure 4b). This platform contains all the tools included in the system, including bioinformatics analysis and enrichment tools, sequence characterization, predictions of biological activities via classification model binaries trained based on the biological activities updated in this new version of Peptipedia, machine learning application methods for building predictive models, and pattern identification methods based on peptide sequence clustering techniques and unsupervised learning algorithms.

Among the new tools incorporated in this latest version of Peptipedia, machine learning methods play an essential role in predicting and studying unknown peptide sequences and training predictive models or pattern recognition.

Only amino acid sequences are required when using classification models of biological activities, and the system will automatically predict the selected biological activities. To do this, the system collects the sequences, numerically represents them according to the pretrained model corresponding to the activity of interest, applies the model, and delivers the responses. A configurable probability threshold parameter is incorporated to customize predictions and provide greater decision control to the user.

The training of predictive models can be carried out using the corresponding tool implemented in PeptiTools (see Section S5 of Supplementary data for more details). These models are based on numerical representation of sequences, so they do not employ structural techniques or feature engineering, only allowing the application of pretrained models and encodings based on physicochemical properties. Besides, different configurations for preprocessing and training models are available, including (i) selecting the algorithm to train the model, (ii) validation strategies, and (iii) standardization and dimensionality reduction applications. Once the model is trained, the results are displayed on the platform, which varies depending on the type of response used to train the model, indicating specific information such as scatter plots for regression models and sensitivity–specificity analysis with confusion matrices for classification models.

When applying clustering strategies for pattern recognition, the tool requires input sequences, the selection of a numerical representation strategy, and the choice of methodology. The tool processes the entered configuration, applies the approach, and generates the results, displaying the characterized groups and a summary of the process (see section S5 of Supplementary data for more details).

### How to use Peptipedia v2.0? Examples and advice to improve the user-friendly experience

Peptipedia v2.0 stands out as a comprehensive resource for the search and analysis of peptide sequences with reported biological activities in the literature. Beyond serving as a powerful search site, this platform facilitates the description of peptides from phylogenetic, structural, descriptive, and functional perspectives, making it an integrated system for both searching and characterizing sequences.

Firstly, Peptipedia v2.0 optimizes the search for peptide sequences by integrating various data sources and features, allowing users to apply different filters, explore results, and download them in an accessible format. The ability to combine filters and narrow the search range enables users to refine their queries based on specific descriptors, such as sequence length or isoelectric point. Peptipedia v2.0 also facilitates searches by filtering for canonical residues and biological activities, both reported and predicted. It is worth noting that including sequences with predicted biological activities increases the number of results (see Figure S6 in Section S5 of the Supplementary data for more details). However, it is important to consider the potential margin of error associated with predictive models.

Secondly, Peptipedia v2.0 allows for a detailed study of peptide sequences through the application of various bioinformatics and predictive tools. For example, the platform facilitates the phylogenetic evaluation of sequences, as well as the prediction of Gene Ontology terms and Pfam domains. Additionally, it offers tools for predicting structural properties and estimating physicochemical properties, enabling a complete characterization of sequences of interest. By utilizing predictive models, Peptipedia v2.0 also allows for the *in silico* identification of peptides with potential biological activities, making it useful in the characterization of unknown peptides obtained from sequencing processes, to predict both biological activities and physicochemical properties.

A third application is related to the use of the machine learning tools available in Peptipedia v2.0. These tools allow the application of clustering methods to identify patterns and the use of predictive modelling strategies to train classification systems or predict desired properties by the user. An example would be training an antiviral peptide classification model specific to viruses of the Retroviridae family. The user can select the type of numerical representation, the algorithm to apply, and the validation strategy. Peptipedia v2.0 handles the model training, generating a performance report and providing a section to facilitate the use of the model in predicting new sequences.

By bringing together various tools in a single web platform, Peptipedia v2.0 not only allows for the search and identification of peptides reported in the literature but also provides simplified access to powerful bioinformatics tools without the need for complex installations on a personal computer. In this way, the challenges associated with the installation and execution of tools, as well as user requirements, software components, and operating system compatibility, are eliminated, as everything is integrated into an accessible and efficient web platform for the study of peptides and their biological activities.

## Conclusions

This work introduces Peptipedia v2.0, a significant update to our previously reported peptide database. Peptipedia v2.0 integrates data from over 70 sources, compiling over 100 000 peptides with reported biological activity and over 3.8 million peptide sequences without reported biological activity. The functional biological activity tree has been updated, increasing the number of biological activities and refining the hierarchical structure to facilitate the efficient study of peptide functions.

In this new version of Peptipedia, all registered peptides are characterized using enrichment analysis approaches, integrating gene ontology term predictions, secondary structure evaluation, and functional domain analysis. Additionally, various physicochemical and thermodynamic properties are estimated for each peptide sequence, enhancing the knowledge base of Peptipedia v2.0. The database also includes patents, references, and pharmacological properties related to the peptide sequences.

The classification models have been enhanced with the updated functional biological activity tree. A combination of numerical representation strategies based on embedding approaches through pretrained models and classical supervised learning algorithms was used to train the classification models, achieving an average sensitivity and specificity of 87% across more than 95 evaluated models. These models were utilized to identify potential therapeutic peptides and predict the functional biological activity of the 3.8 million previously uncharacterized peptide sequences.

Furthermore, Peptipedia v2.0 incorporates new services and tools, including enrichment analysis, statistical evaluation, and machine learning–based strategies, to facilitate the study of peptide sequences. By updating the database and developing these tools, Peptipedia v2.0 positions itself as one of the most comprehensive public repositories for peptide research, playing a crucial role in various research areas.

## Supplementary Material

baae113_Supp

## Data Availability

Source code and datasets are available on the GitHub repository: (i) Peptipedia v2.0 database https://github.com/ProteinEngineering-PESB2/Peptipedia and (ii)Peptipedia v2.0 tools: https://github.com/ProteinEngineering-PESB2/Peptitools. The ETL process scripts are located in https://github.com/ProteinEngineering-PESB2/PeptipediaParser. The user-friendly web platform is publicly accessible through https://app.peptipedia.cl/ for non-commercial uses, licensed under a Creative Commons CC BY-NC-ND 4.0 license. The Peptipedia v2.0 database is available for non-commercial use and is licensed under an ODbl license. The SQL dump file, the peptide sequences with the biological activities, and the peptide sequences with the predicted biological activities applying the trained predictive models are available on https://drive.google.com/drive/folders/1IDNhWmROMfdpgj6ADunBgVb0YBBBaJui?usp=drive_link.
